# OCT Findings in Patients with Methamphetamine Use Disorder

**DOI:** 10.3390/jpm13020308

**Published:** 2023-02-10

**Authors:** Şüheda Kaya, Mehmet Kaan Kaya

**Affiliations:** 1 Elazıg Mental Health Hospital, Elazig 23200, Turkey; 2 Ophthalmology Clinic, Universal Göz Hospital, Elazig 23040, Turkey

**Keywords:** methamphetamine use disorder, optic coherence tomography, retinal nerve fiber layer thickness

## Abstract

Purpose: In the present study, the purpose was to examine the results of optical coherence tomography (OCT) measurements in patients diagnosed with methamphetamine use disorder (MUD) by comparing them with healthy controls. Materials and Methods: A total of 114 eyes were evaluated in this study (27 patients and 30 control group participants). After detailed biomicroscopic examinations of all participants by the same ophthalmologist, both eyes were evaluated by OCT. The retinal nerve fiber layer thickness (RNFL) and macular thickness were calculated from OCT. Results: No statistically significant differences were detected between the demographic data of the patient and control groups (*p* > 0.05). When OCT findings were evaluated, macular thickness and volume were not different between the groups (*p* > 0.05). With respect to RNFL, the left eye superior, inferior, temporal, and nasal quadrants, as well as the left eye’s total measurements were found to be thicker than those of controls (*p* < 0.05). In both eyes, the left eye nasal quadrant and APIS total score were negatively correlated, the total RNLF measurement of the right eye and APIS motivation subscale score were negatively correlated, central macular thickness and the APIS motivation subscale score were positively correlated, and the APIS substance use characteristics subscale score and left eye temporal quadrant RNLF measurement were positively correlated. Conclusion: Our study is the first to evaluate addiction severity and OCT findings in MUD. However, this study needs to be supported by further studies so that OCT findings, which can be used as an effective method for demonstrating possible neurodegeneration in methamphetamine use disorder, gain importance.

## 1. Introduction

Methamphetamine is a potent psychostimulant for the central nervous system (CNS), which causes the release and inhibits the reuptake of monoamine neurotransmitters, including dopamine, norepinephrine, and serotonin [1]. Methamphetamine, a derivative of amphetamine, stimulates the CNS more potently than amphetamine. Methamphetamine is mostly drunk or snorted; less often it is swallowed or injected orally. Clinical manifestations of methamphetamine use include increased energy and alertness, euphoria, sympathetic nervous system activation, decreased need for sleep, weight loss, dry mouth and irritability leading to caries, anxiety, aggression, panic, suspiciousness and/or paranoia, hallucinations, executive dysfunction and symptoms such as memory impairment. Methamphetamine use disorder (MUD) is a worldwide public healthcare issue. The acute complications of methamphetamine are more dangerous than those of other stimulants because of its long-term neurotoxicity and high addictive potential. Methamphetamine was first synthesized from ephedrine by Japanese chemist Nagayoshi Nagai in 1893, and it was synthesized in crystalline form by Japanese chemist Akira Ogata in 1919 [2]. The fact that these substances can be easily synthesized in the laboratory by using precursors led to the emergence of different types. Methamphetamine was first used as a weight loss drug and as a medical treatment for narcolepsy, asthma, depression, and also to keep troops awake during World War II [3]. However, due to its euphoric and energizing side effects, its legal production was stopped at that time as abuse became widespread. Today, amphetamine-derived drugs are used in the treatment of attention deficit hyperactivity disorder (ADHD) [4].

Methamphetamine use is associated with psychiatric disorders such as depression, psychosis, anxiety, insomnia, pulmonary and cardiovascular pathology, risk of developing Parkinson’s disease, cognitive disorders, and other health problems. 

In recent years, methamphetamine abuse has been on the rise, making it the second most commonly used illegal substance worldwide. It was reported that 0.7% of the population aged 12 and over in the USA (or 1.9 million people) use it [5]. A recent study [6] reported a 23% increase in methamphetamine positivity in the urine samples from various health and clinical settings in the USA since March 2020, and therefore, an increase in methamphetamine abuse during the COVID-19 pandemic.

Methamphetamine is an indirectly acting sympathomimetic amine that easily crosses the blood–brain barrier and is distributed throughout the brain. It causes an increase in dopamine, serotonin and noradrenaline in the central and peripheral nervous system. Methamphetamine enhances monoaminergic neurotransmission through two main mechanisms of action: releasing monoamines from storage vesicles into the cytosol, and reversing the action of monoamine transporters (DAT, NET, SERT, and VMAT2). The overstimulation of monoaminergic pathways leads to severe dysfunction in various brain regions, including the prefrontal cortex and hippocampus. It also causes neuronal damage by stimulating the glial cells in the brain and causing the release of proinflammatory cytokines and chemokines [7].

Optical coherence tomography (OCT) is a medical non-invasive imaging method developed by Huang in 1991 to measure the coherence of light reflected on the tissue and displays biological tissue layers with high-resolution sections [8,9]. The OCT technique was first described by Dr. Fujimoto, a professor of physics at the Massachusetts Institute of Technology, and his team. The first use of OCT in ophthalmology was at the Boston Tufts University New England Eye Center, where Dr. Puliafito and Dr. Schuman applied it to the study of the anterior segment, retinal diseases and glaucoma [10]. It is a harmless application that does not contain radiation and works by sending infrared light at a wavelength of ~800 nm to the tissues and measuring the reflection delay time and intensity, allowing the imaging of tissues and their pathologies. It is similar to ultrasonography in terms of its working logic, but it allows one to take cross-sectional images with much higher resolution. Its application is easy, painless and fast; it is applied without touching the patient’s eye. Central macular thickness (CMT), mean macular thickness (MMT), mean macular volume (MMV), retinal optic disc, and retinal nerve fiber layer (RNFL) thickness can be measured with OCT [8]. In RNFL thickness measurements made with OCT, it has been reported that with every 10 years of aging, there is a decrease of approximately 1 μm in mean RNFL thickness [11]. In normal population evaluations with different spectral OCTs, mean RNFL thickness was also reported to be in the range of 90–113 μm. The retina layer is a part of the central nervous system and is an important region where neural degeneration can be detected. OCT is prominent in the diagnosis and follow-up of retinal diseases and the number of studies examining retinal and neural network changes in neurodegenerative diseases such as Alzheimer’s and Parkinson’s Diseases is increasing [12,13]. As for psychiatric diseases, a relationship was detected between neural degeneration in the retina layer of patients with schizophrenia and the severity of the disease, findings which were then also identified in patients with major depressive disorder and obsessive-compulsive disorder [10,14]. There are very few studies reporting retinal neural loss using OCT in methamphetamine use disorder [15,16]. For this reason, the purpose of the present study was to compare the RNLF, MMT, and CMT measurements of patients diagnosed with methamphetamine use disorder with those of healthy controls by using OCT, in an effort to contribute to the data in the literature.

## 2. Method

The approval was obtained from the Fırat University Non-Interventional Clinical Research Ethics Committee (Number 2022/12-17, dated 20 October 2022). The study was completed after obtaining Ethics Committee Approval in line with the Declaration of Helsinki principles.

Patients diagnosed with MUD according to DSM 5 criteria who applied to the Elazig Mental Health and Diseases Hospital Alcohol and Substance Addiction Treatment Center for inpatient treatment within the period approved by the ethics committee were included.

The inclusion criteria for patients were:

1. No DSM 5 diagnosis other than MUD (other substance use disorders, psychotic disorders, mood disorders, ADHD);
2. No previous eye disease (those with intraocular pressure greater than 20 mmHg and axial sphere outside 20–24 mm length, retinal pathologies, cataract, glaucoma, optic neuritis, spherical and cylindrical refractive errors greater than +/− 1.00 diopters, uveitis, history of corneal diseases, ocular trauma, and neurological disorders were not included in the study);
3. Absence of a neurological diagnosis;
4. Being between the ages of 18 and 65;
5. Give written consent to participate in the study.

The inclusion criteria for the control group were:

1. Absence of a psychiatric diagnosis that meets DSM 5 criteria and absence of a neurological diagnosis;
2. No previous eye disease (those with intraocular pressure greater than 20 mmHg and axial sphere outside 20–24 mm length, retinal pathologies, cataract, glaucoma, optic neuritis, spherical and cylindrical refractive errors greater than +/− 1.00 diopters, uveitis, history of corneal diseases, ocular trauma, and neurological disorders were not included in the study);
3. Being between the ages of 18 and 65;
4. Give written consent to participate in the study.

This study was planned as a case–control study. The patients and the control group were informed about the study by the responsible psychiatrist (Ş.K) and ophthalmologist (M.K.K). Those who met the criteria and gave consent were included. Because all of the individuals in the patient group were male, the control group was also selected from males. The control group was formed randomly from healthy subjects who gave consent and who applied to the hospital due to reasons such as job application and who did not have any psychological, neurological, or eye diseases. The SCID-5-CV (Structured Clinical Interview for DSM-5) [17] was administered to all participants by the same psychiatrist. Patients who met the criteria were asked to fill out a sociodemographic data form and APIS form. Only the sociodemographic data form was filled in the control group. Written informed consent was obtained to participate in the study. After this stage, biometric eye measurements and OCT were performed by the ophthalmologist (M.K.K). Tomographic evaluations were performed on the selected eye using the OCT (Huvitz OCT-1F) without pupil dilation. The HOCT-1F uses a diode beam source of 840 nm and has an axial resolution of 6 µm with a scanning speed of 68,000 axial scans per second; this system uses an intelligent fixation system that avoids artifacts from ocular movements. OCT images had an SSI > 7, which is the optimal reference value for the quality of the image. A 45° color fundus photograph (12 megapixels) was also taken simultaneously with the tomographic evaluation without pupil dilation. The minimum sample size required to detect a significant difference using this test should be at least 17 in each group (34 in total), considering type error (alpha) of 0.05, power (1-beta) of 0.8, effect size of 1.02 and two-sided alternative hypothesis (H1) [18]. A total of 35 patients with a diagnosis of MUD were included in the study. However, 8 people were excluded from the study because diseases such as glaucoma and optic trauma were detected in their eye examinations. In total, 57 people, 27 in the patient group and 30 in the control group, were included in the study.

### 2.1. Sociodemographic and Clinical Data Form

We prepared a sociodemographic and clinical data form that was in line with our clinical experience and the information obtained from the reviewed sources and considered the aims of the study for use in this study. This semi-structured form included sociodemographic information such as age, gender, marital status, educational status, occupation, place of residence, economic status, and family structure.

### 2.2. Addiction Profile Index (APIS) Self-Report Scale

The Addiction Profile Index (API) is a self-report questionnaire that consists of 37 items and 5 subscales. In our study, the Turkish form, whose validity and reliability was evaluated by Ögel et al., was used for the patient and control groups [19,20]. The API self-report form consisted of five subscales: substance use characteristics (SUC), diagnostic characteristics (DC), impacts on life (IOL), severe desire (SD), and motivation to quit the substance (MQS). Subscale scores are evaluated separately, and the score for the whole scale is obtained by weighting the subscale. As a total score, the highest is 20, the lowest is 0, and 12 points or less indicates low addiction severity, 12–14 points indicate medium addiction severity, and 14 points or more indicates high addiction severity.

### 2.3. OCT Measurements

**Figure 1:**
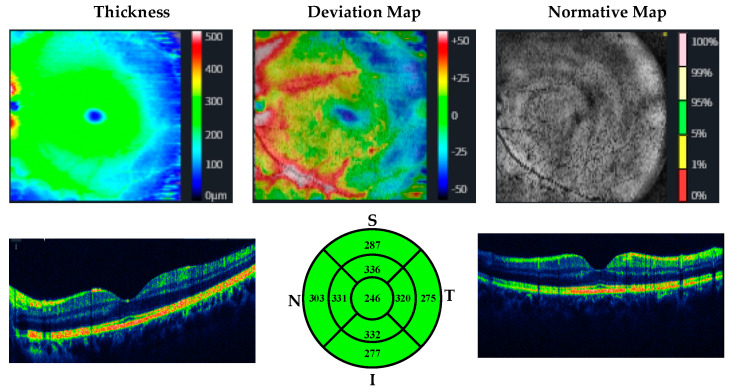
OCT analysis of the macula in a healthy participant.

**Figure 2:**
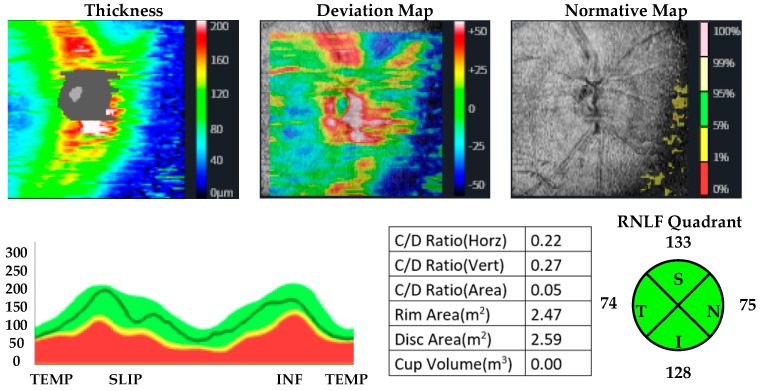
OCT analysis of the RNLF in a patient with methamphetamine use disorder.

**Figure 3:**
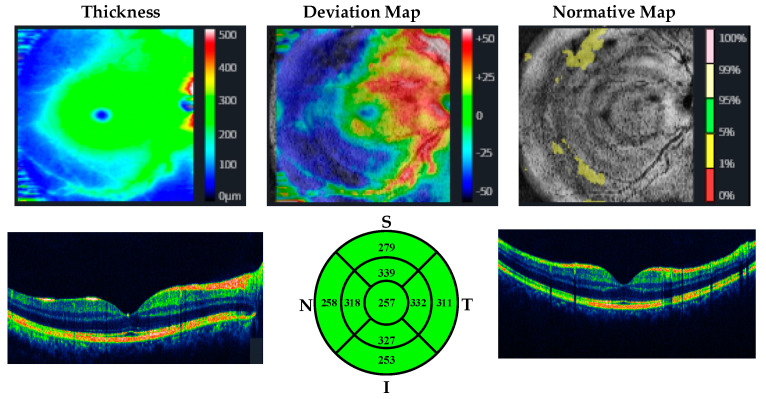
OCT analysis of the macula in a patient with methamphetamine use disorder.

**Figure 4:**
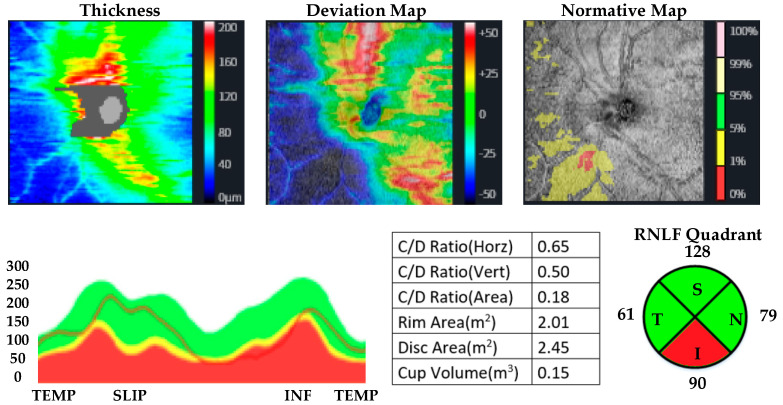
OCT analysis of the RNLF in a healthy participant.

OCT measurements were performed by the same ophthalmologist for all participants. CMT, MMT, and RNLF were measured. The protocol used for measuring RNFL thickness was the optic disc cube with circumpapillary RNFL thickness measurements calculated from a circular scan with a diameter of 3.46 mm (10.87 mm in length) automatically placed around the optic disc. The macular thickness was acquired using the macular cube 512 × 128 protocol, where a 6 × 6 mm macular area is covered, collecting 512 × 128 scans (horizontal and vertical). The axial lengths of the patients and controls were measured by using a biometer. Huvitz 1-F was used as the optical coherence tomography device. OCT images of participants with methamphetamine use disorder and healthy participants are given in Figure 1, Figure 2, Figure 3 and Figure 4.

### 2.4. Statistical Method

The SPSS 27.0 package program was used in the analysis of the data. Mean ± Standard Deviation, median (min-max), frequency, and percentage values were used in the descriptive statistics of the data. The distribution of the variables was evaluated with the Shapiro–Wilk Test. Independent samples *t*-tests and Mann–Whitney U-tests were used in the analysis of the quantitative data from two independent groups. The Pearson and Spearman correlation analyses were used to determine the relationship between two independent variables. The significance level was taken as 0.05.

## 3. Results

According to the DSM-5 diagnostic criteria, 27 individuals with MUD and 30 individuals in the healthy control group (a total of 57 participants) were included in the study. Since all of the patient groups were male, the control group was also male. No differences were detected between the patient and control groups in terms of age, gender, marital status, education level, and working status (*p* < 0.05). The mean age of the patient group was 28 ± 6.4, and that of the control group was 22.5 ± 10.6 (Table 1). The patients’ OCT measurements were taken on the first day of hospitalization before the start of treatment. The mean age of first substance use in the patient group was 19.96 ± 7.23. The average years of methamphetamine use were 8.04 ± 0.83 years and the daily dose was calculated as 0.8 ± 0.10 g. The MUD patient group and healthy controls had 1 pack/day of cigarette use.

The total RNLF measurement results in both eyes were significantly thicker (*p* < 0.001) in the patient group and the RNLF measurement results in the left eye’s superior and inferior quadrants were significantly thicker in the patient group than in the control group (*p* < 0.05). The temporal quadrant RNLF measurements of both eyes were significantly thicker in the patient group than in the control group (*p* < 0.05). The nasal quadrant RNLF measurements in both eyes of the patient group were significantly thicker than those of the control group (*p* < 0.05). No significant differences were detected between the groups in terms of the superior and inferior quadrant RNLF measurements of the right eye (*p* > 0.05). No significant differences were detected between the groups in terms of middle and central macular thicknesses (*p* > 0.05) (Table 2).

With respect to the subscales of the APIS self-assessment questionnaire, the average score for the substance use characteristic subscale was 2.74 ± 1.66, the mean score for the diagnosis subscale was 14.6 ± 5.6, the mean score for the impacts on life subscale was 29.7 ± 6.4, the mean score for the severe desire subscale was 8.4 ± 4.3, and the mean score for the motivation subscale was 11.03 ± 1.5. The mean total score of the APIS was found to be 12.58 ± 3.06.

A negative correlation was detected between the left eye nasal quadrant and the APIS total score when evaluating the correlation between the APIS total score and eye measurements. A negative correlation was found between the motivation subscale and the total RNLF measurement in the right eye, and a positive correlation was found between the central macular thicknesses of both eyes. A positive correlation was detected between the substance use characteristics subscale and the RNLF of the left eye temporal quadrant. (Table 3).

## 4. Discussion

A total of 57 people were included in the present study, in which RNLF and MMT measurements taken by OCT were compared between patients with methamphetamine use disorder and healthy controls. No significant differences were detected between the patient and control groups in terms of demographic data (*p* > 0.05). In the optical coherence tomography results, the RNFL was found to be thicker in participants with MUD than in healthy controls in some lower quadrants. Macular thickness was not different between groups. Certain sub-quadrants and APIS scores were correlated, providing evidence for a relationship between addiction severity and other variables of addiction and OCT results. 

The retina is an important part of the central nervous system (CNS), and is seen as the “window of the brain”. It develops from the anterior part of the neural tube, like our brain, in the early developmental stage. With this similarity in its development, it is considered that a change in brain function may be reflected in the retina [21]. With this mechanism in mind, studies conducted with OCT in individuals with psychiatric diseases have gained momentum in recent years [10,12,13,14,15,16]. However, a slightly more limited number of papers have studied addiction and OCT measurements. The results reported are contradictory [15,16,22,23,24,25]. In a study in which OCT measurements were measured in patients with alcohol use disorder, RNLF thicknesses were not different from those of healthy controls [22]. In another study that was conducted with patients diagnosed with alcohol use disorder, all quadrants of trace RNLF were found to be thinner than those of healthy controls [24]. In a study conducted with the opiate use disorder group, RNLF thickening was observed in the total quadrant and superior quadrant [23]. In another study, although the RNLF was higher in both eyes than in the superior quadrant controls, the nasal quadrant was thinner in the right eye [25]. In the first study that examined OCT findings in patients with methamphetamine use disorder, Talebnejad et al. found RNLF thicknesses lower than those of healthy controls [15]. In one of the other studies that were conducted with MUD patients, no difference was detected between patients and healthy controls. In one patient group, minimal thinning was found in the superior and temporal quadrants [16,26]. In an animal study that considered three groups of mice (mice injected with a high or low dose of methamphetamine, as well as healthy mice), it was shown that increased chemokines resulted in damage to the retina in the mouse group injected with high-dose methamphetamine [27]. In another study in which pregnant rats were injected with methamphetamine, no difference was found in terms of neurotransmitters that could cause retinal development or retinal damage compared to controls [28]. In our results, the RNLF thicknesses of the temporal and nasal quadrant, as well as total measurements of the eyes, were found to be higher than those of healthy controls. In addition, measurements of the left eye superior and inferior quadrants were higher than those of healthy controls. The neurotransmitter dopamine plays roles in the basic modulation of the retina and the development of addiction [18,29]. In studies conducted on rodents, it was shown that psychostimulant substances such as amphetamine/methamphetamine increase extracellular dopamine release in acute intake [30,31]. However, in chronic, long-term exposure, they experience dopamine depletion at the striatal level [32]. The same thing was found in people with amphetamine/methamphetamine use disorder. In post-mortem analyses, it is noteworthy that striatal dopamine levels were low in MUD patients. Both low dopamine levels and increased dopamine receptor sensitivity were reported [31,33]. With this mechanism in mind, it is thought that the RNLF layer may be increased in patients with MUD.

Macular thickness is another area that was examined in the present study. In the literature, macular thickness was not evaluated in the first studies conducted with MUD patients [15]. In another study, it was observed that there was thinning in certain quadrants of the macula [26]. In addition, in another study conducted in our country, no differences were found in terms of macular thickness between patients with MUD and healthy controls [16]. Our results were similar to those of that study (macular thickness did not differ in any quadrant between controls and patients with MUD).

Finally, our patient group with MUD was evaluated as moderately addicted based on APIS total scores. The left eye nasal quadrant and APIS total score were negatively correlated, the right eye total RNLF measurement and APIS motivation subscale score were negatively correlated, and central macular thickness and the APIS motivation subscale score were positively correlated in both eyes. Finally, a positive correlation was detected between the APIS substance use characteristics subscale score and the RNLF thickness of the temporal quadrant of the left eye. The present study was the first study in which disease severity was evaluated by examining the MUD patient group and OCT measurements. In other studies, parameters such as roughly how many years of MUD and whether or not participants received psychiatric treatment were examined. In another study conducted with this calculation, methamphetamine usage per month was reported as 43.91 ± 23.23 and daily use was 0.94 ± 0.90 g [16]. In another study, monthly use was 6.97 ± 2.61 years and daily use was calculated as 0.007 ± 0.003 g [26]. In our patient group, the monthly use was calculated as 8.04 ± 0.83 years, and the daily dose was calculated as 0.8 ± 0.10 g. Our patient group was moderately addicted according to the APIS scores. Although the data from the literature are similar in terms of the duration of use and daily use doses, the results obtained in our study were inconsistent with the results in the literature, and the compatibility of our data with the literature was low. Although the data obtained here regarding OCT findings in patients with MUD—an area with a limited number of studies in the literature—make important contributions to the literature, there is a need for more comprehensive studies in this area.

Our results should be evaluated by considering some limitations. The first of the limitations was the relatively low number of patients. The unequal distribution between the genders was another limitation. Further studies, including simultaneous evaluation of retrobulbar ocular blood flow parameters and OCT angiography of the optic nerve and longitudinal and neuroimaging techniques, are required in larger sample groups for the findings obtained in the present study to gain importance.

In conclusion, a thickened RNFL was detected in certain quadrants in patients with MUD. Macular thickness was not different between patients and controls. On the other hand, OCT measurements were associated with the severity of addiction of the patients in some quadrants. In light of our findings, when evaluating patients in the addiction group and planning treatment, it may be recommended to consider visual findings and possible neurodegeneration. Considering the limitations of our study, more comprehensive studies should be planned. In addition, these diseases can be detected by artificial intelligence techniques. Moreover, the detection of diseases with OCT, EEG, ECG, and MRI can be accomplished with artificial intelligence techniques [34,35,36,37,38,39,40,41,42,43,44,45,46,47]. In the future, addiction detection studies using artificial intelligence techniques can be done with OCT images.

## Figures and Tables

**Table 1:** The demographic characteristics of the patient and control groups.

	MUD Patient Group		Healthy Control Group		*p*
	N	%	N	%	
Gender (Male/Female)	27/0	100	30/0	100	>0.05
**Marital Status**					
Married	11	40.7	13	43.3	>0.05
Single	14	51.9	15	50	
Widow(er)/Divorced	2	7.4	2	6.7	
**Educational Status**					
Primary School	5	18.5	4	13.3	>0.05
Secondary School	14	51.9	16	53.3	
High School	5	18.5	6	20	
University	3	11.1	4	13.3	
**Working Status**					
Part-time	4	14.8	6	20	>0.05
Full-time	10	37	13	43.3	
Unemployed	13	48.1	11	36.6	

The chi-square test was used in the calculations.

**Table 2:** Retinal nerve fiber layer thickness evaluated by optical coherence tomography.

		MUD Patient Group (N = 27) (Mean ± sd)	Healthy Control Group (N = 30) (Mean ± sd)	*p*	d
Retinal nerve fiber layer thickness	**Superior quadrant**				
	Right eye	125.70 ± 10.05	119.00 ± 16.98	0.079 ^a^	d: 0.44, r: 0.219
	Left eye	129.85 ± 9.79	115.53 ± 25.89	**0.002 ^b^**	d: 0.74, r: 0.34
	**Inferior quadrant**				
	Right eye	130.93 ± 12.47	125.77 ± 16.18	0.187 ^a^	d: 0.35, r: 0.17
	Left eye	134.67 ± 15.03	125.53 ± 16.25	**0.032 ^a^**	**d: 0.58, r: 0.27**
	**Temporal quadrant**				
	Right eye	82.67 ± 8.96	64.43 ± 12.10	**<0.001 ^a^**	d: 1.76, r: 0.66
	Left eye	76.70 ± 7.51	64.13 ± 12.17	**<0.001 ^b^**	**d: 1.22, r: 0.52**
	**Nasal quadrant**				
	Right eye	84.81 ± 10.35	76.83 ± 13.13	**0.012 ^b^**	**d: 0.68, r: 0.32**
	Left eye	82.15 ± 11.69	75.23 ± 13.28	**0.019 ^b^**	**d: 0.58, r: 0.27**
	**Total value**				
	Right eye	116.81 ± 9.35	96.50 ± 10.04	**<0.001 ^a^**	**d: 2.10, r: 0.72**
	Left eye	106.30 ± 7.99	96.53 ± 9.93	**<0.001 ^b^**	**d: 1.24, r: 0.52**
Macular thickness	**Central Macular**				
	Right eye	245.26 ± 15.54	249.00 ± 16.92	0.390 ^a^	d: −0.25, r: −0.12
	Left eye	244.33 ± 13.49	247.40 ± 19.09	0.491 ^a^	d: −0.18, r: −0.09
	**Mean Macular**				
	Right eye	280.93 ± 9.61	286.33 ± 13.70	0.094 ^a^	d: −0.53, r: −0.25
	Left eye	283.00 ± 10.21	283.17 ± 13.22	0.958 ^a^	d: −0.08, r: −0.04
APIS	SUC	2.7433 ± 1.66			
	D	14.68 ± 5.68			
	IOL	29.74 ± 6.40			
	SD	8.44 ± 4.39			
	M	11.03 ± 1.55			

^a^ Mann–Whitney U-test and ^b^ independent sampling *t*-test were used in the calculations, d: Cohen’s d value, r: effect size. APIS Total: Addiction Profile Index Scale total score, D: diagnosis subscale, IOL: impact on life subscale, M: motivation subscale, SD: severe desire subscale, SUC: substance use characteristics subscale.

**Table 3:** Analysis of the correlation between APIS score and RNLF and macula thicknesses.

		SUC	D	IOL	SD	M	APIS
RNLF thickness							
**Superior**							
Right eye	r	0.107	0.361	0.071	−0.006	−0.065	0.205
Left eye	r	0.340	0.108	0.080	0.178	−0.119	0.247
**Inferior**							
Right eye	r	0.291	−0.028	0.212	0.234	−0.157	0.120
Left eye	r	−0.122	−0.083	0.285	−0.200	−0.200	−0.080
**Temporal**							
Right eye	r	−0.123	−0.074	0.107	0.032	0.053	−0.061
Left eye	**r**	0.475 *	0.064	0.253	0.056	0.158	0.242
**Nasal**							
Right eye	r	0.187	−0.060	0.030	0.031	−0.075	−0.011
Left eye	r	−0.288	−0.180	−0.100	−0.300	−0.343	−0.394 *
**Total value**							
Right eye	r	0.134	0.059	0.055	−0.114	−0.390 *	−0.080
Left eye	r	−0.245	−0.200	0.040	−0.193	0.060	−0.200
**Macular thickness**							
**Central**							
Right eye	r	−0.205	−0.063	−0.168	−0.108	0.506 **	−0.095
Left eye	r	−0.147	−0.015	−0.044	−0.028	0.550 **	0.019
**Mean**							
Right eye	r	−0.169	−0.301	−0.072	−0.178	0.284	−0.201
Left eye	r	−0.105	−0.308	−0.082	−0.074	0.338	−0.169

Pearson and Spearman correlation analysis was used in the calculations. The values in the table are “r” values. * *p* < 0.05, ** *p* < 0.01. APIS Total: Addiction Profile Index Scale total score, D: diagnosis subscale, IOL: impact on life subscale, M: motivation subscale, SD: severe desire subscale, SUC: substance use characteristics subscale.

## Data Availability

The data are not publicly available.
